# Inflammatory Stress Increases Hepatic CD36 Translational Efficiency via Activation of the mTOR Signalling Pathway

**DOI:** 10.1371/journal.pone.0103071

**Published:** 2014-07-21

**Authors:** Chuan Wang, Lin Hu, Lei Zhao, Ping Yang, John F. Moorhead, Zac Varghese, Yaxi Chen, Xiong Z. Ruan

**Affiliations:** 1 Centre for Lipid Research, Key Laboratory of Molecular Biology on Infectious Diseases, Ministry of Education, the Second Affiliated Hospital of Chongqing Medical University, Chongqing, China; 2 John Moorhead Research Laboratory, Centre for Nephrology, University College London (UCL) Medical School, Royal Free Campus, University College London, United Kingdom; Institute of Medical Research A Lanari-IDIM, University of Buenos Aires-National Council of Scientific and Technological Research (CONICET), Argentina

## Abstract

Inflammatory stress is an independent risk factor for the development of non-alcoholic fatty liver disease (NAFLD). Although CD36 is known to facilitate long-chain fatty acid uptake and contributes to NAFLD progression, the mechanisms that link inflammatory stress to hepatic CD36 expression and steatosis remain unclear. As the mammalian target of rapamycin (mTOR) signalling pathway is involved in CD36 translational activation, this study was undertaken to investigate whether inflammatory stress enhances hepatic CD36 expression via mTOR signalling pathway and the underlying mechanisms. To induce inflammatory stress, we used tumour necrosis factor alpha (TNF-α) and interleukin-6 (IL-6) stimulation of the human hepatoblastoma HepG2 cells *in vitro* and casein injection in C57BL/6J mice *in vivo*. The data showed that inflammatory stress increased hepatic CD36 protein levels but had no effect on mRNA expression. A protein degradation assay revealed that CD36 protein stability was not different between HepG2 cells treated with or without TNF-α or IL-6. A polysomal analysis indicated that CD36 translational efficiency was significantly increased by inflammatory stress. Additionally, inflammatory stress enhanced the phosphorylation of mTOR and its downstream translational regulators including p70S6K, 4E-BP1 and eIF4E. Rapamycin, an mTOR-specific inhibitor, reduced the phosphorylation of mTOR signalling pathway and decreased the CD36 translational efficiency and protein level even under inflammatory stress resulting in the alleviation of inflammatory stress-induced hepatic lipid accumulation. This study demonstrates that the activation of the mTOR signalling pathway increases hepatic CD36 translational efficiency, resulting in increased CD36 protein expression under inflammatory stress.

## Introduction

Non-alcoholic fatty liver disease (NAFLD) encompasses a spectrum of liver diseases without significant alcohol consumption, ranging from non-alcoholic simple fatty liver (NAFL) to non-alcoholic steatohepatitis (NASH), hepatic fibrosis and cirrhosis. NAFLD has become the leading cause of chronic liver injury in developed countries. Numerous experimental and clinical studies have demonstrated that inflammatory stress is an independent risk factor in NAFLD [1]–[3]. Both *in vitro* and *in vivo* models have shown that inflammatory stress promotes hepatic lipid accumulation by up-regulating or down-regulating important lipometabolic proteins or enzymes [4]–[6].

Donnelly recently demonstrated that about 59% of liver fatty acids in NAFLD patients is derived from the circulation, 26% from *de novo* liver fatty acids synthesis and only 15% from the diet [7]. When insulin does not suppress triglyceride (TG) hydrolysis in adipose tissue, fatty acids are released into the circulation to form albumin/fatty acid complexes, which transport into hepatocytes by either passive transport or fatty acid transporting proteins (FATPs) including CD36 (also called ‘fatty acid translocase (FAT)’), plasma membrane fatty acid binding protein (FABPpm) and caveolin. Of these CD36 plays an important role in hepatic fatty acids transport. CD36 is a transmembrane glycoprotein which is a type B scavenger receptor expressed in various cells associated with energy metabolism, including adipocytes [8], pancreatic beta cells [9], skeletal myocytes and hepatocytes [10]. This multifunctional receptor has been studied extensively with regard to its role in facilitating the uptake of long-chain fatty acids and oxidised low-density lipoproteins, which are involved in the aetiology of such metabolic disorders as diabetes [11], atherosclerosis [12] and NAFLD [13]. Overexpression of CD36 aggravates fatty acid uptake and triglyceride storage in human hepatoma cells and the livers of C57BL6 mice [14], [15]. In patients with NAFLD, CD36 up-regulation is significantly associated with liver fat accumulation [16]. These findings suggest that hepatic CD36 expression is closely related to hepatic steatosis.

The term ‘metabolic inflammation’ has emerged from the close association of metabolic dysfunction and long-term chronic inflammatory stress in NAFLD. It is characterized by increased serum levels of C-reactive protein and pro-inflammatory cytokines such as TNF-α, IL-6 and IL-8. Studies have shown that inflammatory stress up-regulates CD36 expression in vessels [17], but the effect of inflammatory stress on hepatic CD36 regulation and the mechanisms that control hepatic fatty acids trafficking remains unclear.

In general, the expression of CD36 can be regulated at the transcriptional, translational, or post-translational levels, In the promotion of hepatic steatosis, CD36 is a transcriptional target of orphan nuclear receptors including liver X receptor (LXR), pregnane X receptor (PXR) and peroxisome proliferator-activated receptor γ (PPARγ) [18]. However, our pilot study demonstrated that inflammatory stress enhanced hepatic CD36 protein level without concomitant increase in the expression of CD36 mRNA revealing a surprisingly imbalance between protein and mRNA. It implies that inflammatory stress may alter post-transcriptional expression of hepatic CD36.

The activation of mammalian target of rapamycin (mTOR) in response to inflammatory stress is involved in the progression of metabolic syndrome [19], [20]. mTOR is a widely distributed and highly conserved serine/threonine kinase that plays a crucial role in the regulation of proliferation, angiogenesis [21], [22] and protein translation including CD36 [23], [24]. mTOR integrates stimulating signals and phosphorylates eukaryotic initiation factor 4E-binding protein 1 (4E-BP1) and p70 ribosomal protein S6 kinase (p70S6K) [25], [26]. Phosphorylation of 4E-BP1 inhibits its binding to eukaryotic initiation factor 4E (eIF4E) [27], [28], which directs the 5′ cap structure containing 7-methylguanosine triphosphate to the 40S ribosomal subunit and promotes more efficient translation during the initiation phase of translation [29]. Another branch of the mTOR signalling pathway, p70S6K is involved in translational regulation by phosphorylating the S6 protein of the 40S ribosomal subunit and enhancing the translation of mRNAs characterized by an oligopyrimidine tract in the 5′ terminal [30]. However, the precise regulatory mechanism of mTOR signalling pathway-mediated hepatic CD36 protein expression under inflammatory stress is largely unknown.

The present study was undertaken to investigate whether inflammatory stress enhances hepatic CD36 expression via the mTOR signalling pathway-mediated translational regulation of CD36 in HepG2 cells and C57BL/6J mice. Furthermore, we assessed the effect of rapamycin, an mTOR-specific inhibitor, on hepatic CD36 translational efficiency and steatosis under inflammatory stress *in vitro* and *in vivo*.

## Materials and Methods

### Cell Culture

The human hepatoblastoma cell line HepG2 was obtained from the American Type Culture Collection. The experiments were performed in a serum-free medium containing Dulbecco’s modified Eagle medium-high glucose, 0.2% bovine serum albumin (BSA), 0.04 mmol/L palmitate. The cells were subjected to 10 ng/mL rapamycin (Sangon Biotech, Shanghai, China) or inflammatory cytokines by loading 25 ng/mL tumour necrosis factor alpha (TNF-α, PeproTech, Rocky Hill, NJ, USA) or 20 ng/mL interleukin-6 (IL-6, Sinobio, Shanghai, China) for 24 hours.

### Animal Model

The animal care and experimental procedures were approved by the Animal Care Committee of Chongqing Medical University. Six-to eight-week-old male C57BL/6J mice were fed a normal chow diet (NCD, Research Diets, New Brunswick, NJ, USA) and randomly divided into three groups: NCD, NCD plus 0.5 mL 10% casein injection, and NCD plus 0.5 mL 10% casein and rapamycin (2 mg/kg body weight) injection. Subcutaneous injections were performed once daily, Monday through Friday. The mice were sacrificed under deep anesthesia with pentobarbital sodium (60 mg/kg body weight) at 14 weeks after the first injection.

### Total RNA Isolation and Real-Time PCR

Total RNA was isolated from cultured HepG2 cells and frozen liver tissues using the RNAiso Kit (Takara, Dalian, Liaoning, China) according to the manufacturer’s protocol. Real-time PCR was performed to amplify CD36 and β-actin with SYBR Green dye and the following specific primers: human CD36 (forward) 5′-AAATAAACCTCCTTGGCCTGA-3′ and (reverse) 5′-GCAACAAACATCACCACACC-3′; mouse CD36 (forward) 5′-TTGAAGGCATTCCCACGTATC-3′ and (reverse) 5′-CGGACCCGTTGGCAAA-3′; human β-actin (forward) 5′-CCTGGCACCCAGCACAAT-3′ and (reverse) 5′-GCCGATCCACACGGAGTA-3′; mouse β-actin (forward) 5′-CGATGCCCTGAGGCTCTTT-3′ and (reverse) 5′-TGGATGCCACAGGATTCCAT-3′.

### Western Blotting

Identical amounts of protein from cultured HepG2 cells and frozen liver tissue homogenates were separated by SDS-PAGE, transferred to PVDF membranes, and immunoblotted with primary antibodies followed by incubation with an HRP-conjugated secondary antibody. Finally detection procedures were performed using Immobilon Western Chemiluminescent HRP Substrate (Millipore, Temecula, CA, USA). Primary antibodies against the following proteins were used: CD36, total p70S6K, p-p70S6K (Thr 421/Ser 424), total 4E-BP1, p-4E-BP1 (Ser 65/Thr 70), total eIF4E, p-eIF4E (Ser 209) and β-actin (Santa Cruz, Dallas, TX, USA); total mTOR (Millipore, Temecula, CA, USA); and p-mTOR (phospho S2448) (Abcam, Cambridge, UK).

### Protein Degradation Assay

For the CD36 protein stability studies, HepG2 cells were incubated in a serum-free medium containing 14 mg/L cycloheximide (CHX, Genview, Florida, USA), an inhibitor of protein synthesis, in the presence or absence of 25 ng/mL TNF-α or 20 ng/mL IL-6. Total protein was isolated from the cells, and the CD36 and β-actin protein levels were detected by western blotting at the indicated time.

### Polysome Analysis

A polysomal analysis was performed as previously described [31], [32] with some modifications. HepG2 cells and liver tissues were harvested and polysomes were extracted with 0.3 mL of low-salt buffer (LSB, 20 mM Tris-HCl, pH 7.4, containing 10 mM NaCl and 3 mM MgCl_2_) prior to homogenization in 0.1 mL of LSB containing 1.2% (v/v) Triton X-100 and 0.2 M sucrose. The lysate was applied to a 7%–47% linear sucrose gradient and subjected to ultracentrifugation at 36 000×g for two hours at 4°C. The absorbance at 254 nm was determined using a photometer, and the positions of CD36, 28S rRNA, 18S rRNA and β-actin were detected by semiquantitative PCR using the primer sequences listed in Table 1.

**Table 1:** Primers for semiquantitative PCR.

Gene	Primer sequences
Human CD36	Forward: 5′-GAGAGAACTGTTATGGGGCTAT-3′
	Reverse: 5′-TTCAACTGGAGAGGCAAAGG-3′
Human 28S rRNA	Forward: 5′-TTGAAAATCCGGGGGAGAG-3′
	Reverse: 5′-ACATTGTTCCAACATGCCAG-3′
Human 18S rRNA	Forward: 5′-CAGCCACCCGAGATTGAGCA-3′
	Reverse: 5′-TAGTAGCGACGGGCGGTGTG-3′
Human β-actin	Forward: 5′-AGCGAGCATCCCCCAAAGTT-3′
	Reverse: 5′-GGGCACGAAGGCTCATCATT-3′
Mouse CD36	Forward: 5′-GAGCCATCTTTGAGCCTTCA-3′
	Reverse: 5′-TCAGATCCGAACACAGCGTA-3′
Mouse 28S rRNA	Forward: 5′-TTGAAAATCCGGGGGAGAG-3′
	Reverse: 5′-ACATTGTTCCAACATGCCAG-3′
Mouse 18S rRNA	Forward: 5′-AGGGGAGAGCGGGTAAGAGA-3′
	Reverse: 5′-GGACAGGACTAGGCGGAACA-3′
Mouse β-actin	Forward: 5′-GTCCCTCACCCTCCCAAAAG-3′
	Reverse: 5′-GCTGCCTCAACACCTCAACCC-3′

### Measurement of Free Fatty Acid (FFA) Uptake

HepG2 cells were transiently transfected with CD36 siRNA or negative control siRNA, CD36 siRNA (Sense: 5′-GGCUGUGUUUGGAGGUAUUCUTT-3′, Antisense: 5′-AGAAUACCUCCAAACACAGCCTT-3′) or negative control siRNA (Sense: 5′-UUCUCCGAACGUGUCACGUTT-3′, Antisense: 5′-ACGUGACACGUUCGGAGAATT-3′). 1×10^6^ cells were transfected using of the electroporation as described in our previous publication [33]. 24 hours after transfection, the transfected cells were treated by different conditions. The treated cells were harvested for further experiments.

To determine time-dependent FFA uptake, HepG2 cells were washed three times with PBS and further incubated with PBS containing 0.02 mmol/L fluorescein isothiocyanate (FITC)-labeled hexadecanoic acid (Invitrogen, Carlsbad, California, USA). The FITC fluorescence was visualized using a fluorescence microscopy system at the indicated time.

To quantitatively detect the content of FFA uptake in HepG2 cells, a flow cytometry analysis was performed as previously described [34] with some modifications. HepG2 cells were washed three times with FACS buffer (PBS containing 0.2% BSA) and further incubated with FACS buffer containing 0.5 µmol/L FITC-labeled hexadecanoic acid in the presence or absence of 25 µmol/L of excess of palmitate for 2 hours at 37°C, which competitively blocks FITC-labeled hexadecanoic acid uptake. The cells were washed three times with FACS buffer, fixed with 4% paraformaldehyde solution for 20 minutes, and analyzed by flow cytometry. The specific uptake of FITC-labeled hexadecanoic acid via receptors was calculated using the fluorescence intensities in the absence of 25 µmol/L palmitate minus the fluorescence intensities in the presence of 25 µmol/L palmitate to eliminate the influence of non-receptor-mediated FFA uptake.

### Morphological Examination

Lipid accumulation in the HepG2 cells and livers of C57BL/6J mice was evaluated by Oil Red O staining. The samples were fixed with 5% formalin solution in PBS for 30 minutes, stained with Oil Red O for 30 minutes, and counter-stained with haematoxylin for another five minutes. The results were examined by light microscopy.

### Quantitative Measurement of Intracellular FFA and TG Levels

Intracellular FFA and TG levels of the HepG2 cells and livers of C57BL/6J mice were measured using an ELISA kit (CUSABIO, Wuhan, Hubei, China) and enzymatic assay (DONGOU, Wenzhou, Zhejiang, China), respectively. The lipids were extracted in solvent (dipropylmethane/isopropanol = 2∶3.5) and dried under vacuum, and then the concentrations of FFA and TG were analysed using a standard curve and normalised to the total protein from the cells or liver tissues.

### Statistical Analysis

All the data are reported as the mean ± standard deviation. Comparison between groups was performed with one-way ANOVA followed by Q-test using SPSS17.0 software. A *P* value of less than 0.05 was considered significant.

## Results

### Inflammatory Stress Increases Hepatic CD36 Protein Level but has No Effect on CD36 mRNA Expression

**Figure 1:**
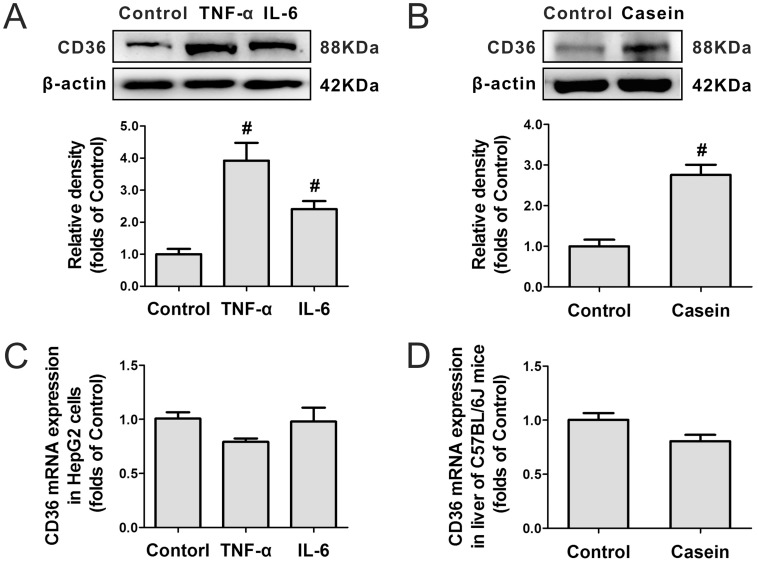
Effect of inflammatory stress on hepatic CD36 protein and mRNA expression. HepG2 cells were pre-incubated for 24 hours in serum-free medium and then incubated for another 24 hours in serum-free medium (Control) or medium containing 25 ng/mL TNF-α (TNF-α) or 20 ng/mL IL-6 (IL-6). C57BL/6J mice were fed a normal chow diet (Control) or a normal chow diet plus casein injection (Casein) for 14 weeks. The protein level of CD36 in the cells (A) and livers (B) was examined by western blotting, and β-actin served as the internal reference. The mRNA expression of CD36 in the cells (C) and livers (D) was determined by real-time PCR, and β-actin served as the housekeeping gene. The results are depicted as the mean ± SD from at least three separate experiments. *P<0.05 versus the control, ^#^ *P*<0.01 versus the control.

Inflammatory stress significantly enhanced CD36 protein expression in the HepG2 cells (Figure 1A) and livers of C57BL/6J mice (Figure 1B). Interestingly, there was no obvious change in the expression of CD36 mRNA *in vitro* (Figure 1C) or *in vivo* (Figure 1D), suggesting that the increase in CD36 protein expression occurred at translational or post-translational level.

### Inflammatory Stress Enhances CD36 Translational Efficiency but has No Effect on CD36 Protein Stability

**Figure 2:**
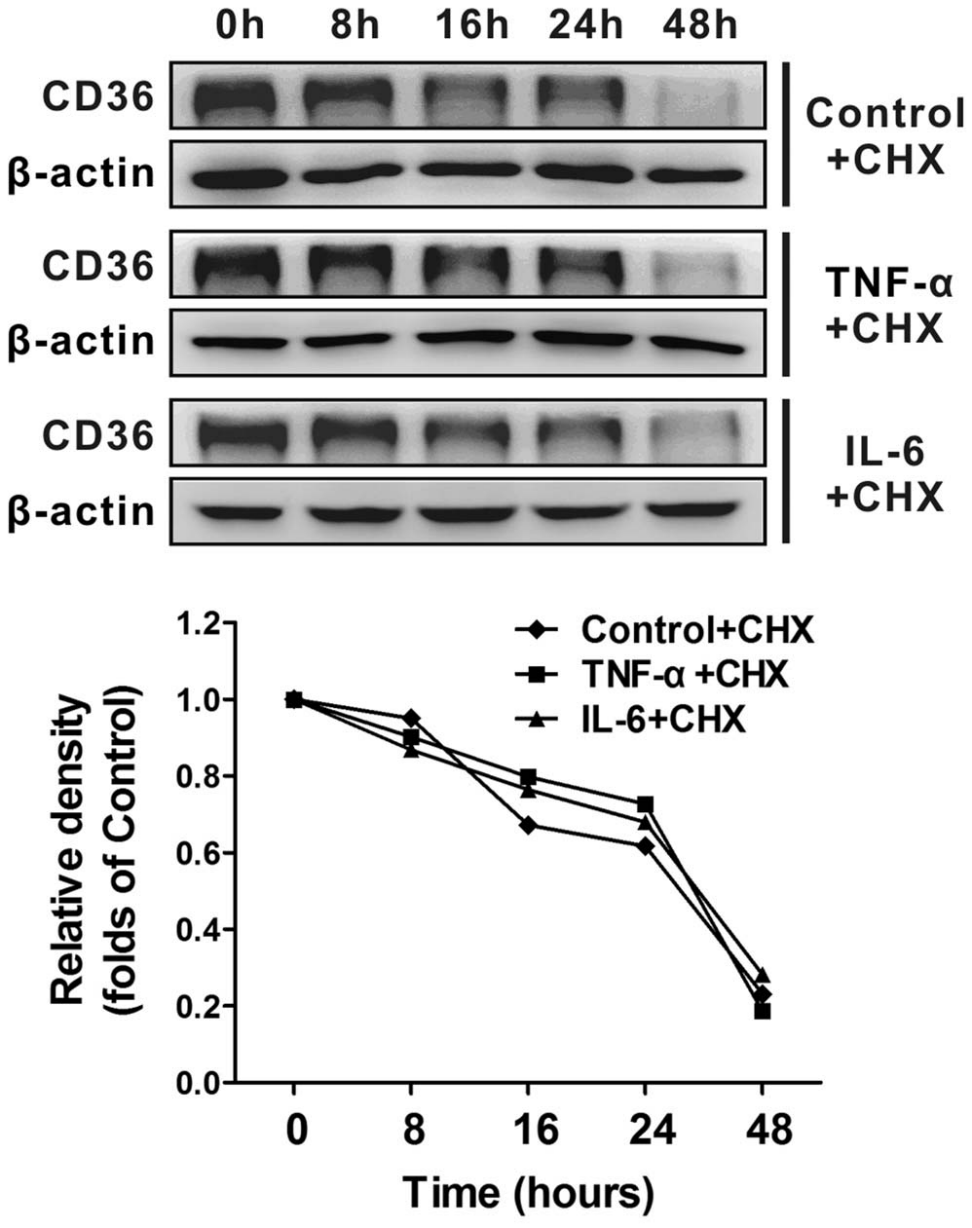
Effect of inflammatory cytokines on hepatic CD36 protein stability. HepG2 cells were pre-incubated in serum-free medium for 24 hours; 14 mg/L CHX in the presence or absence of 25 ng/mL TNF-α or 20 ng/mL IL-6 was then added to the medium, and the cells were incubated for the indicated time. The CD36 protein levels were analysed by western blotting and were normalised to β-actin. The results are depicted as the mean ± SD from three separate experiments. The statistical significance was set at *P*<0.05.

Because increase in the post-transcriptional protein level may be due to the enhancement of protein stability or translational efficiency, we examined the effect of inflammatory cytokines on the degradation of the CD36 protein. CHX blocks peptidyl transferase activity and was used to stop protein synthesis for the estimation of the half-life of the protein. We measured the CD36 protein using western blotting after 0–48 hours of CHX treatment in the presence or absence of TNF-α or IL-6. There was no obvious difference in the CD36 protein half-life in HepG2 cells in the presence or absence of inflammatory cytokines (Figure 2), indicating that inflammatory stress had no effect on the degradation of the CD36 protein.

**Figure 3:**
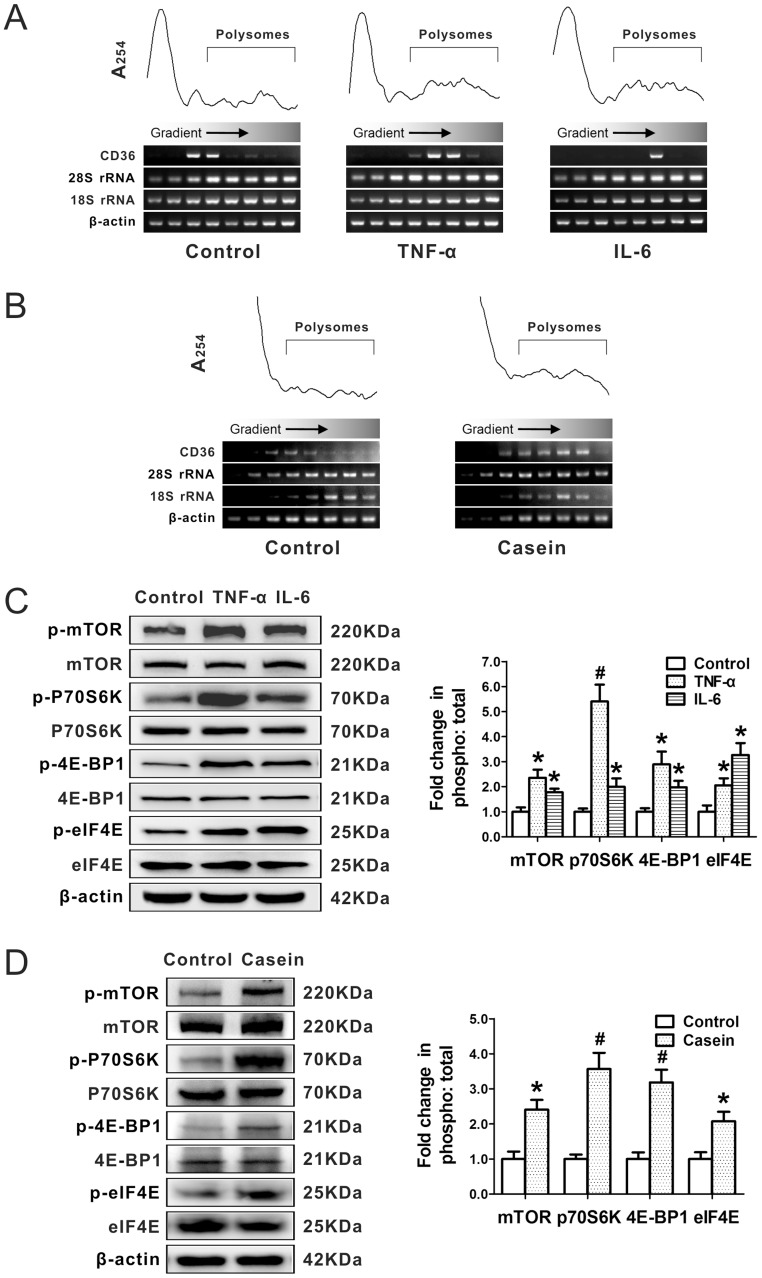
Effect of inflammatory stress on CD36 translational efficiency and phosphorylation of the mTOR signalling pathway. HepG2 cells were pre-incubated for 24 hours in serum-free medium and then incubated for another 24 hours in serum-free medium (Control) or medium containing 25 ng/mL TNF-α (TNF-α) or 20 ng/mL IL-6 (IL-6). C57BL/6J mice were fed a normal chow diet (Control) or a normal chow diet plus casein injection (Casein) for 14 weeks. A polysomal analysis was performed to detect the CD36 translational efficiency in the cells (A) and livers (B). The absorbance at 254 nm was given for those conditions, and the positions of CD36, 28S rRNA, 18S rRNA, and β-actin were detected by semiquantitative PCR. Western blotting analyses were performed for p-mTOR (phospho S2448), total mTOR, p-p70S6K (Thr 421/Ser 424), total p70S6K, p-4E-BP1 (Ser 65/Thr 70), total 4E-BP1, p-eIF4E (Ser 209), total eIF4E, and β-actin in the cells (C) and livers (D). The relative band intensities of the phosphorylated protein were normalised to that of the total protein. The results are depicted as the mean ± SD from three separate experiments. *P<0.05 versus the control, ^#^ *P*<0.01 versus the control.

To determine whether CD36 translational efficiency is sensitive to inflammatory stress in HepG2 cells and the livers of C57BL/6J mice, we performed a polysome analysis for CD36 in the presence or absence of inflammatory stress. The absorbance at 254 nm showed a separation of polysomes using sucrose density gradient ultracentrifugation, and semiquantitative PCR was performed on the gradient fractions. We found that the CD36 mRNA derived from HepG2 cells treated with inflammatory cytokines (Figure 3A) and the livers of C57BL/6J mice treated with casein injection (Figure 3B) was shifted to heavier fractions, indicating an increased number of ribosomes and the active translation of CD36. These findings suggest that the increased expression of CD36 protein under inflammatory stress was mediated by enhanced translational efficiency.

### The mTOR Signalling Pathway Is Involved in the Translational Regulation of Hepatic CD36

We examined the molecular mechanisms by which inflammatory stress up-regulated CD36 protein expression. The data show that inflammatory stress enhanced the phosphorylation of mTOR, p70S6K, 4E-BP1 and eIF4E in the HepG2 cells (Figure 3C) and livers of C57BL/6J mice (Figure 3D), suggesting that mTOR signalling pathway is involved in increasing the CD36 translational efficiency under inflammatory stress.

**Figure 4:**
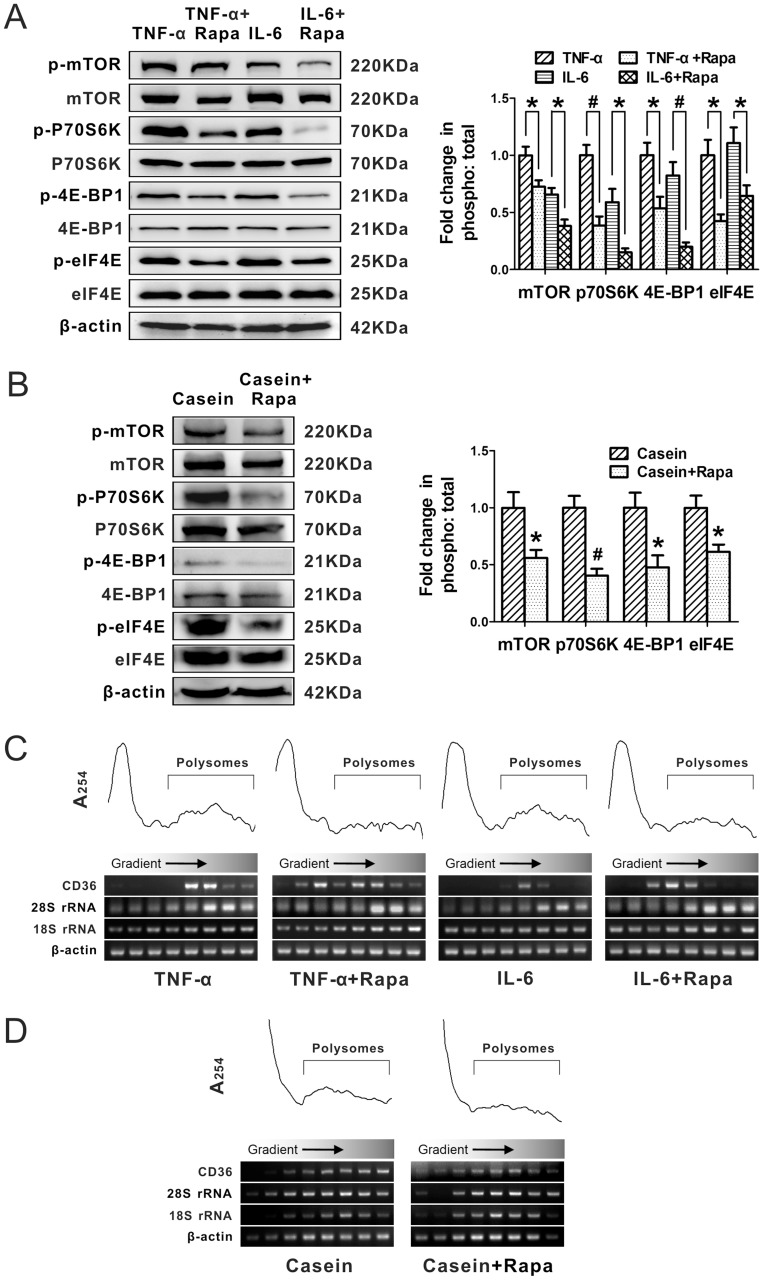
Effect of rapamycin on the phosphorylation of the mTOR signalling pathway and CD36 translational efficiency under inflammatory stress. HepG2 cells were pre-incubated for 24 hours in serum-free medium and then incubated for another 24 hours in serum-free medium containing 25 ng/mL TNF-α (TNF-α) or 25 ng/mL TNF-α plus 10 ng/mL rapamycin (TNF-α+Rapa) or 20 ng/mL IL-6 (IL-6) or 20 ng/mL IL-6 plus 10 ng/mL rapamycin (IL-6+ Rapa). C57BL/6J mice were fed a normal chow diet and received a casein injection (Casein) or casein and rapamycin injection (Casein+Rapa) for 14 weeks. Western blotting analyses were performed for p-mTOR (phospho S2448), total mTOR, p-p70S6K (Thr 421/Ser 424), total p70S6K, p-4E-BP1 (Ser 65/Thr 70), total 4E-BP1, p-eIF4E (Ser 209), total eIF4E, and β-actin in the cells (A) and livers (B). The relative band intensities of the phosphorylated protein were normalised to that of the total protein. A polysomal analysis was performed to detect CD36 translational efficiency in the cells (C) and livers (D). The absorbance at 254 nm was given for those conditions, and the positions of CD36, 28S rRNA, 18S rRNA, and β-actin were detected by semiquantitative PCR. The results are depicted as the mean ± SD from three separate experiments. * denotes a significant difference at *P*<0.05, and ^#^ denotes a significant difference at *P*<0.01.

We further examined the effect of rapamycin, an mTOR-specific inhibitor, on the phosphorylation of mTOR signalling pathway components under inflammatory stress. As expected, rapamycin inhibited the enhanced phosphorylation of mTOR, p70S6K, 4E-BP1 and eIF4E, even under inflammatory stress in the HepG2 cells (Figure 4A) and livers of C57BL/6J mice (Figure 4B). These results indicate that rapamycin decreased the phosphorylation of mTOR signalling pathway components, which was up-regulated by inflammatory stress.

To determine whether the increased CD36 translational efficiency induced by inflammatory stress could be inhibited by rapamycin, we performed a polysome analysis for CD36 under inflammatory stress in the presence or absence of rapamycin. The absorbance at 254 nm showed the separation of polysomes using sucrose density gradient ultracentrifugation, and semiquantitative PCR was performed on the gradient fractions. The data showed that the CD36 mRNA derived from the HepG2 cells (Figure 4C) and livers of C57BL/6J mice (Figure 4D) in the presence of rapamycin was shifted to lighter fractions, suggesting a decreased number of ribosomes and inactive translation of CD36. These results indicate that rapamycin inhibited the enhanced phosphorylation of mTOR signalling pathway components and prevented the ribosomal loading of CD36 mRNA under inflammatory stress.

**Figure 5:**
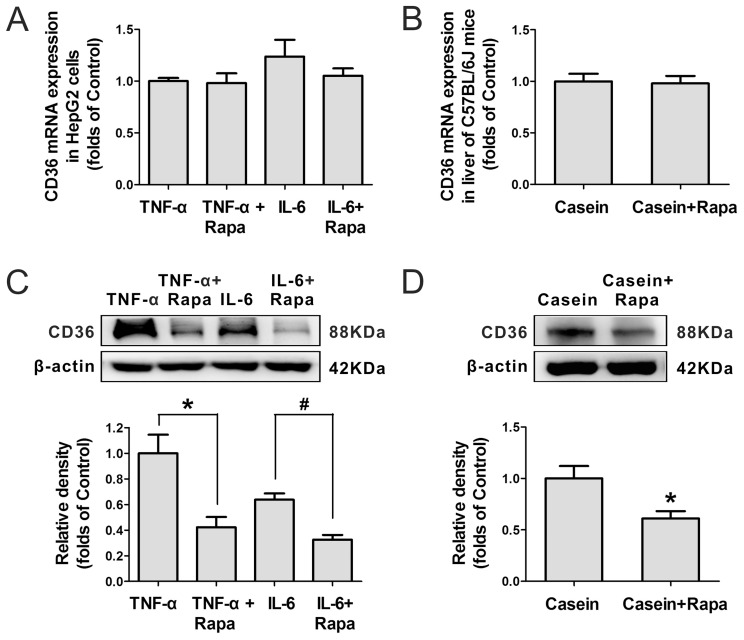
Effect of rapamycin on hepatic CD36 mRNA and protein expression under inflammatory stress. HepG2 cells were pre-incubated for 24 hours in serum-free medium and then incubated for another 24 hours in serum-free medium containing 25 ng/mL TNF-α (TNF-α) or 25 ng/mL TNF-α plus 10 ng/mL rapamycin (TNF-α+Rapa) or 20 ng/mL IL-6 (IL-6) or 20 ng/mL IL-6 plus 10 ng/mL rapamycin (IL-6+ Rapa). C57BL/6J mice were fed a normal chow diet and received a casein injection (Casein) or casein and rapamycin injection (Casein+Rapa) for 14 weeks. The mRNA expression of CD36 in the cells (A) and livers (B) was determined by real-time PCR, and β-actin served as the housekeeping gene. The protein level of CD36 in the cells (C) and livers (D) was examined by western blotting, and β-actin served as the internal reference. The results are depicted as the mean ± SD from at least three separate experiments. * denotes a significant difference at *P*<0.05, and ^#^ denotes a significant difference at *P*<0.01.

We next examined the effect of rapamycin on hepatic CD36 expression under inflammatory stress. Although rapamycin had no effect on CD36 mRNA expression in HepG2 cells (Figure 5A) or livers of C57BL/6J mice (Figure 5B), it led to a significant decrease in CD36 protein expression under inflammatory stress *in vitro* (Figure 5C) and *in vivo* (Figure 5D), suggesting that the CD36 protein expression enhanced by inflammatory stress was mTOR signalling pathway dependent.

### Rapamycin Alleviates Hepatic Lipid Accumulation under Inflammatory Stress

**Figure 6:**
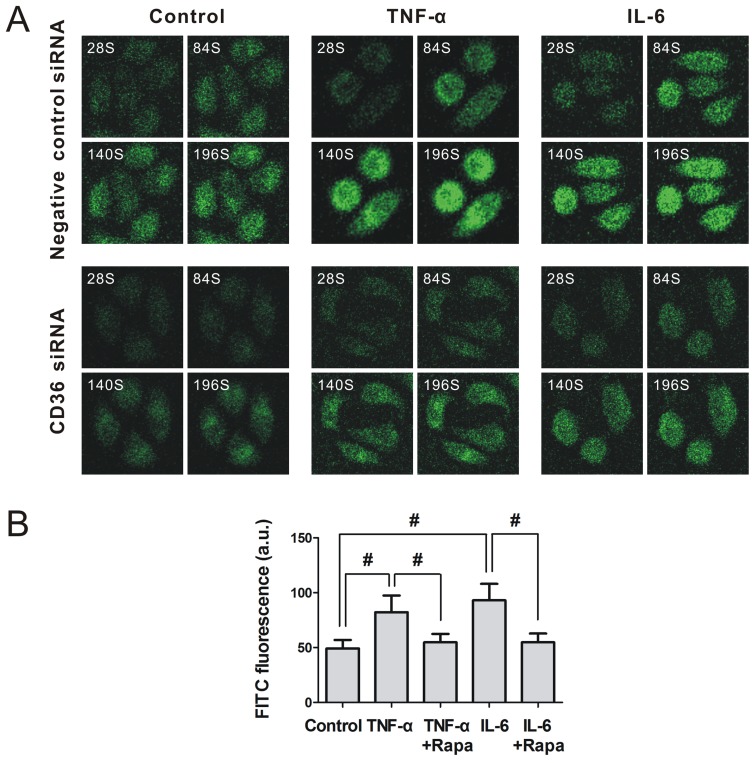
Effect of CD36 siRNA and rapamycin on hepatic FFA uptake under inflammatory stress. HepG2 cells were pre-incubated for 24 hours in serum-free medium and then incubated for another 24 hours in serum-free medium (Control) or medium containing 25 ng/mL TNF-α (TNF-α) or 25 ng/mL TNF-α plus 10 ng/mL rapamycin (TNF-α+Rapa) or 20 ng/mL IL-6 (IL-6) or 20 ng/mL IL-6 plus 10 ng/mL rapamycin (IL-6+ Rapa). (A) The effect of CD36 siRNA on FITC-labeled hexadecanoic acid uptake by HepG2 cells under inflammatory stress was determined using fluorescence microscopy. (B) The effect of rapamycin on FITC-labeled hexadecanoic acid uptake by HepG2 cells under inflammatory stress was detected using flow cytometry. The results are depicted as the mean ± SD from three separate experiments. * denotes a significant difference at *P*<0.05, and ^#^ denotes a significant difference at *P*<0.01.

**Figure 7:**
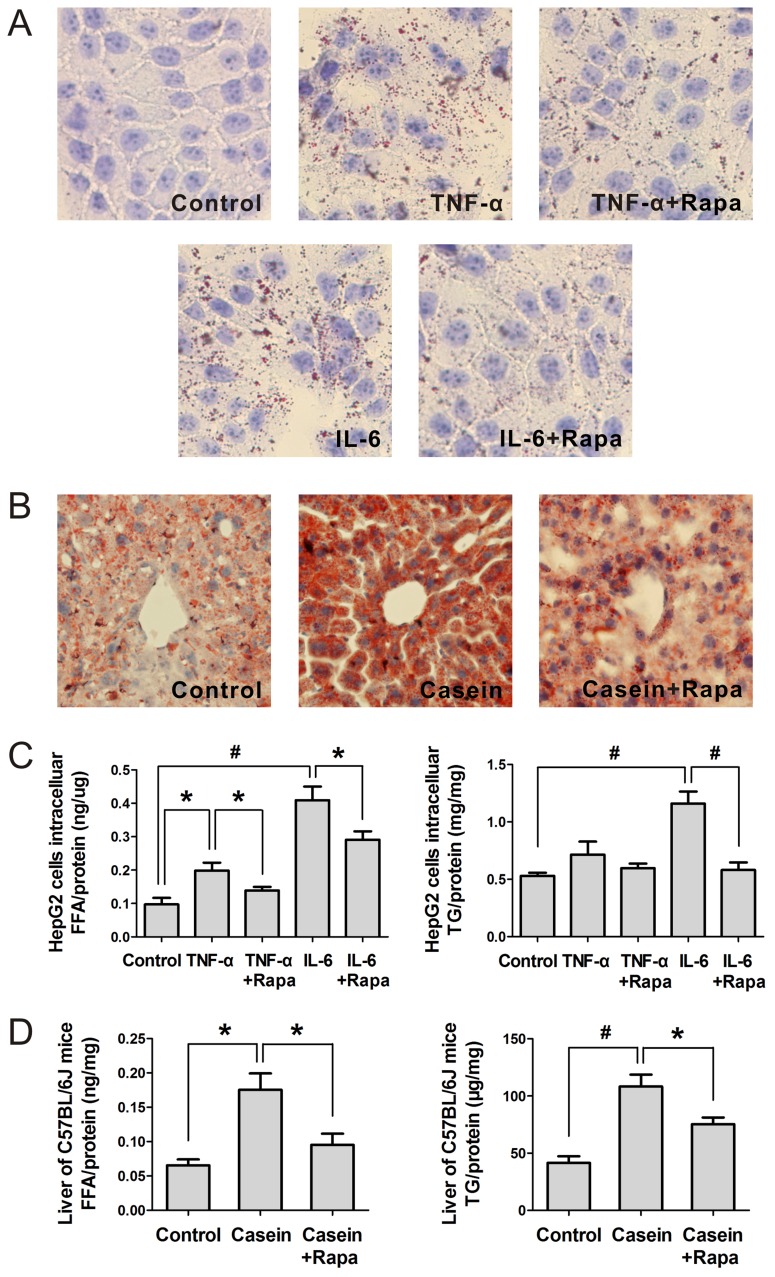
Effect of rapamycin on hepatic lipid accumulation under inflammatory stress. HepG2 cells were pre-incubated for 24 hours in serum-free medium and then incubated for another 24 hours in serum-free medium (Control) or medium containing 25 ng/mL TNF-α (TNF-α) or 25 ng/mL TNF-α plus 10 ng/mL rapamycin (TNF-α+Rapa) or 20 ng/mL IL-6 (IL-6) or 20 ng/mL IL-6 plus 10 ng/mL rapamycin (IL-6+ Rapa). C57BL/6J mice were fed a normal chow diet (Control), a normal chow diet plus casein injection (Casein), or a normal chow diet plus casein and rapamycin injection (Casein+Rapa) for 14 weeks. The lipid accumulation in the cells (A) and livers (B) was observed by Oil Red O staining (original magnification ×400). The concentrations of FFA and TG in the cells (C) and livers (D) were measured as described in the Materials and Methods. The results are depicted as the mean ± SD from at least three separate experiments. * denotes a significant difference at *P*<0.05, and ^#^ denotes a significant difference at *P*<0.01.

The uptake rate of FFA in HepG2 cells was determined by fluorescence microscopy. Inflammatory stress accelerated the uptake of FITC-labeled hexadecanoic acid, which was reduced by knock down of CD36 (Figure 6A), suggesting that CD36 mediated the uptake of FFA. Furthermore, the amount of FFA uptake by HepG2 cells was quantified by flow cytometry, we found that inflammatory stress significantly increased the FITC fluorescence intensities of HepG2 cells, whereas rapamycin reduced the enhanced FITC fluorescence intensities induced by inflammatory stress (Figure 6B). Using Oil Red O staining, we found that inflammatory stress significantly increased hepatic lipid droplet accumulation, whereas rapamycin alleviated the lipid droplet accumulation induced by inflammatory stress in the HepG2 cells (Figure 7A) and livers of C57BL/6J mice (Figure 7B). A quantitative assay for FFA and TG (Figure 7C and 7D) confirmed the results of the Oil Red O staining, suggesting that rapamycin provided a protective role in decreasing hepatic lipid accumulation induced by inflammatory stress *in vitro* and *in vivo*.

## Discussion

Many studies have indicated that hepatic inflammatory stress contributes to the progression of NAFLD, as described by our group [35] and others [36]. Hepatic inflammatory stress is a critical event in lipid accumulation and may exacerbate lipid-mediated hepatocyte injury. In our previous study, chronic systemic inflammation was found to accelerate lipolysis and decelerate lipogenesis in adipose tissues while enhancing lipogenesis in liver tissues, resulting in hepatic ectopic lipid deposition in C57BL/6J mice [4].

In the present study, we used TNF-α or IL-6 treatment and subcutaneous injection of casein to induce inflammatory stress in HepG2 cells and C57BL/6J mice. Pro-inflammatory cytokines such as TNF-α and IL-6 are key inflammatory markers that are able to induce inflammatory stress in cell models. Casein injection induces chronic systemic inflammation that is similar to inflammatory diseases in patients and is commonly used in atherosclerosis and NAFLD models [5], [19].

Our data demonstrate that inflammatory stress increased hepatic CD36 protein level but had no effect on CD36 mRNA expression, suggesting that inflammatory stress disrupts CD36 protein expression at a translational or post-translational level rather than a transcriptional level. This may be because inflammatory stress cannot up-regulate the transcriptional regulators of CD36 including LXR, PXR and PPARγ in liver [6], [37].

We evaluated the protein stability of CD36 in HepG2 cells after inflammatory cytokines treatment using a protein degradation assay because enhanced translational efficiency or prolonged protein half-life could result in an increase in the level of CD36 protein. The data show that the degree of CD36 protein degradation was not significantly different between the HepG2 cells treated with or without either TNF-α or IL-6 indicating that inflammatory stress did not reduce the degradation of the CD36 protein in HepG2 cells.

Translational regulation which is found in various diseases determines final protein levels when transcription is inhibited or silent. Hsieh demonstrated that the translational activation of some key mRNAs which are associated with cancer initiation and metastasis promotes prostate cancer cell migration and invasion [38]. Alexandrov showed that activation of PTEN and Stat3 mRNA translation leads to hepatic insulin resistance [39]. While Griffin demonstrated that high glucose-induced increased translation of CD36 in macrophage promotes atherosclerosis [31]. Studies of protein synthesis typically utilize polysome analysis to explore the protein synthesis and translational efficiency of individual mRNA; examination of the positions of individual mRNA in the sucrose fractions can indicate whether the mRNA translation. Accordingly, we performed a polysome analysis using sucrose density gradient ultracentrifugation to investigate whether inflammatory stress increased CD36 translational efficiency, which might explain an increase in the CD36 protein level. We found that the CD36 mRNA was shifted to heavier fractions under inflammatory stress *in vitro* and *in vivo*, corresponding to increased ribosome loading and active translation of CD36. However, inflammatory stress did not generate general translational activation, as the position of the β-actin ribosome loading profiles in the gradient was not altered. These data indicate that inflammatory stress-enhanced CD36 translational efficiency was due to the presence of more ribosomes encoding this mRNA, resulting in increased levels of CD36 protein.

Furthermore, we investigated the potential mechanisms by which inflammatory stress increased the CD36 translational efficiency at the translational level. Our data show that inflammatory stress enhanced the phosphorylation of mTOR, p70S6K, 4E-BP1 and eIF4E both *in vitro* and *in vivo*. These results indicate that the inflammatory stress-induced activation of mTOR and its downstream translational regulators, including p70S6K, 4E-BP1 and eIF4E, contributed to enhancing CD36 translation initiation, resulting in increases in the level of CD36 protein.

Rapamycin is an mTOR-specific inhibitor that has been used clinically as an immunosuppressant for the prevention of transplant rejection. Numerous studies have demonstrated that rapamycin alleviates hepatic steatosis via inhibiting some important lipid metabolic enzymes including sterol regulatory element binding protein 1c (SREBP1c), SREBP2, fatty acid synthase (Fasn), acetyl-CoA carboxylase (ACC), stearoyl-CoA desaturase-1 (SCD1), and low-density lipoprotein receptor (LDLr), as described by our group [40] and others [41], [42]. Our data demonstrated that rapamycin inhibited the enhanced phosphorylation of the mTOR signalling pathway components induced by inflammatory stress and decreased CD36 translational efficiency, resulting in a reduction in the CD36 protein level. This finding further confirmed that inflammatory stress-enhanced CD36 protein expression was mediated by the mTOR signalling pathway. Furthermore, we found that rapamycin significantly decreased FFA uptake and alleviated hepatic lipid accumulation *in vitro* and *in vivo* overcoming the effects of inflammatory stress and providing a protective role in alleviating hepatic steatosis. These results demonstrate that inflammatory stress significantly increased FFA uptake, which was reduced by rapamycin, suggesting that FFA uptake via CD36 expression was the one of mechanisms for intracellular lipid accumulation in addition to the lipogenesis.

In conclusion, our findings both *in vitro* and *in vivo* demonstrated that inflammatory stress enhanced hepatic CD36 expression at the translational level, but not the transcriptional level, and activated the mTOR signalling pathway, thereby initiating a translation initiation signal that resulted in increased CD36 protein expression. Rapamycin prevented the lipid accumulation induced by inflammatory stress through the inhibition of the mTOR signalling pathway and CD36 translational efficiency. These results may represent a new molecular mechanism for hepatic steatosis and provide additional evidence for the therapeutic treatment of NAFLD using inhibitors of mTOR in patients with metabolic syndrome.
